# Unraveling the roles of cerebrospinal fluid-contacting neurons

**DOI:** 10.7554/eLife.87054

**Published:** 2023-03-24

**Authors:** Claire Wyart

**Affiliations:** 1 Paris Brain Institute (ICM), Sorbonne Université, INSERM U1127, UMR CNRS 7225 Paris France

**Keywords:** cerebrospinal fluid-contacting neuron, spinal cord, neural circuit, locomotion, proprioception, interoception, PKD2L1, cerebrospinal fluid, mechanosensation, Mouse

## Abstract

Sensory neurons previously shown to optimize speed and balance in fish by providing information about the curvature of the spine show similar morphology and connectivity in mice.

**Related research article** Nakamura Y, Kurabe M, Matsumoto M, Sato T, Miytashita S, Hoshina K, Kamiya Y, Tainaka K, Matsuzawa H, Ohno N, Ueno M. 2023. Cerebrospinal fluid-contacting neuron tracing reveals structural and functional connectivity for locomotion in the mouse spinal cord. *eLife* **12**:e83108. doi: 10.7554/eLife.83108.

With the flick of a fin, limb or wing, vertebrates are able to quickly move and adjust the position of their body, so they can swim towards food, walk through their territory or fly away from danger. To do so, they need to accurately detect ever-changing constraints, for example by identifying possible obstacles around them. Yet locomotion cannot rely only on vision, as this sense fails to detect mechanical stimuli such as external physical pressures, the animal’s own body posture or movement, and the inner forces generated by muscle contractions. Instead, this information is provided through mechanoreception.

In the 1930s, Sir Charles Sherrington and Sir John Eccles pioneered the investigation of mechanosensory feedback in vertebrates (Eccles and Sherrington, 1930). They established that this process takes place via the peripheral nervous system, which relays mechanical information from limbs and other body parts back to cells in the spinal cord that modulate motor contraction. Since then, it has been assumed that perception of posture and movement in vertebrates mainly relies on peripheral mechanosensory feedback. However, recent work in fish and amphibians has started to uncover a central mechanosensory pathway, which lies right within the spinal cord and is tasked with detecting changes in the curvature of the spine.

The spinal cord is formed of bundles of neurons that extend their axons to various parts of the body. At the core of the spinal cord runs a central canal filled with the same cerebrospinal fluid (or CSF) that cushions the brain inside the skull. Within this canal is a group of CSF-contacting neurons (CSF-cNs for short) equipped with small ‘hairs’ – actin-based microvilli and one cilium – that allow them to detect mechanical changes in their environment (Böhm et al., 2016; Jalalvand et al., 2016; Sternberg et al., 2018). Experiments artificially activating these neurons confirmed that they participate in locomotion (Wyart et al., 2009). More recent work has shown that CSF-cNs, which can inhibit the activity of motor neurons and excitatory premotor interneurons, help to increase speed and improve balance during fast movement by connecting with neural cells involved in regulating motor output (Wu et al., 2021).

So far, these results on mechanosensation in the spinal cord have mainly been confined to studies in zebrafish and lampreys; whether this sensorimotor pathway is also relevant the locomotion of land mammals has only recently started to be explored. Although CSF-cNs are conserved in the spinal cord of mice and macaques (Djenoune et al., 2014), these animals collect mechanosensory feedback from their body differently compared to fish: a mouse, for example, would use information from the position of each joint in its limbs, or from the way its paws contact the ground. Now, in eLife, Masaki Ueno from Nigata University and co-workers – including Yuka Nakamura as first author – report that CSF-cN projections and roles in mouse locomotion are similar to those of lamprey and zebrafish (Nakamura et al., 2023). These results complement recently published findings from a study investigating the same questions (Gerstmann et al., 2022).

Nakamura et al. (who are based at various institutes in Japan) first developed an elegant method that allowed them to individually label CSF-CNs and their projections. The technique relies on the fact that these cells express an ion channel known as PKD2L1 (Djenoune et al., 2014). The team genetically engineered mice in which only PKD2L1-carrying cells could produce a fluorescent reporter, which was introduced to the neurons via a viral vector injected in the CSF. These experiments and the work by Gerstmann et al. revealed that, as previously reported in fish, the axons of CSF-cNs project onto the ventral funiculus and synapse onto motor neurons and excitatory premotor interneurons. Strikingly, both bodies of work also discovered that CSF-cNs formed synapses with each other, allowing these cells to form a recurrent circuit.

Next, Nakamura et al. investigated the functional role of CSF-cNs by selectively inactivating these cells in mice that were then placed on a treadmill. As a result, the animals took fewer steps per minute and therefore could not run as fast; in fish, similar manipulations resulted in a decrease in swimming speeds and tail beat frequency (Böhm et al., 2016; Wu et al., 2021). Inactivating CSF-CNs also led to reduced performances in motor and coordination tests (Gerstmann et al., 2022). These observations suggest that fine control of balance during movement may also be impaired in mice, as was previously recorded in fish (Böhm et al., 2016; Hubbard et al., 2016; Wu et al., 2021).

Overall, the work by Nakamura et al. adds to accumulating evidence that the functional morphology, projection patterns and roles of CSF-cNs are conserved within vertebrates (Figure 1). This study, as well as the work by Gerstmann et al., did not investigate the projection patterns and connectivity of CSF-cNs in the hindbrain, which will be an exciting avenue to pursue in the future.

**Figure 1.**
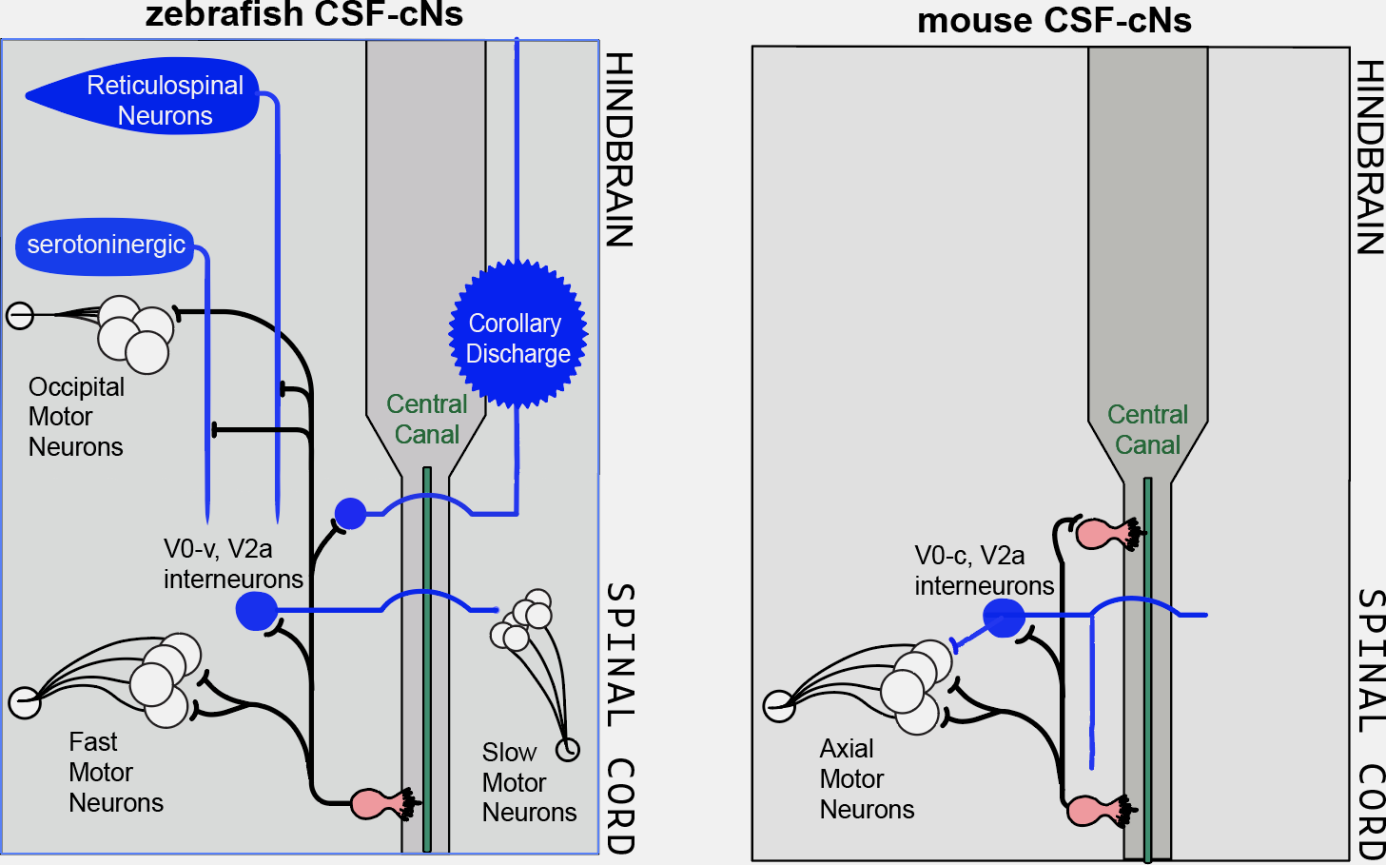
Cerebrospinal-contacting neurons share common functional morphology and neuronal targets in the spinal cord of mice and fish, where they help to optimize balance and locomotion. Cerebrospinal-contacting neurons (CSF-cNs; pink) in the central canal (green) of the spinal cord (dark grey) improve balance and locomotion by sensing mechanical stimuli and sending this information via their axons (black lines) to neurons that control motor outputs. (Left) As described in Wu et al., 2021, in zebrafish, the CSF-cNs connect to various groups of spinal cord cells involved in locomotion, such as excitatory premotor and sensory V0-v and V2a interneurons (blue circles), fast and slow motor neurons (white circles with black outline; bottom), reticulospinal neurons (which relay locomotion-initiating signals from the brain) as well as serotoninergic neurons acting as neuromodulators. Some of the CSF-cNs also project onto groups of cells in the hindbrain, such as occipital motor neurons. Finally, CSF-cNs project onto large sensory interneurons which likely convey information of a corollary discharge (the process by which the brain can be informed of impending movement) back to the hindbrain. (Right) Nakamura et al. showed that, similar to fish, the axons of the CSF-cNs in mice project to motor neurons and pre-motor excitatory interneurons, but also onto other CSF-cNs. This is in agreement with another recently published study (Gerstmann et al., 2022).

Another central question that requires further investigation is whether CSF-cNs sense spinal curvature in mammals, like they do in fish. The transparency and genetic accessibility of zebrafish has enabled scientists to dissect precisely how CSF-cNs operate to sense spinal curvature in these animals. When trunk and head muscles on one side contract, the bending of the spinal cord puts under tension the Reissner fiber, a long polymer running through the central canal (Orts-Del’Immagine et al., 2020). On the concave side (typically the side where the muscles are contracting), the cilia which extend from the CSF-cNs are brought into contact with the Reissner fiber, enabling asymmetric information on the position of the spine to be relayed to motor neural circuits and neurons that control this musculature. This central sensory pathway therefore allows fish to adjust their posture based on the position of their spine, and not just information from their limbs and other body parts. Future studies will tell if these cells also work with the Reissner fiber to act as mechanosensors in the spinal cord of mice (Orts-Del’Immagine et al., 2020).

## References

[bib1] Böhm UL, Prendergast A, Djenoune L, Nunes Figueiredo S, Gomez J, Stokes C, Kaiser S, Suster M, Kawakami K, Charpentier M, Concordet JP, Rio JP, Del Bene F, Wyart C (2016). CSF-contacting neurons regulate locomotion by relaying mechanical stimuli to spinal circuits. Nature Communications.

[bib2] Djenoune L, Khabou H, Joubert F, Quan FB, Nunes Figueiredo S, Bodineau L, Del Bene F, Burcklé C, Tostivint H, Wyart C (2014). Investigation of spinal cerebrospinal fluid-contacting neurons expressing PKD2L1: evidence for a conserved system from fish to primates. Frontiers in Neuroanatomy.

[bib3] Eccles JC, Sherrington CS (1930). Reflex summation in the ipsilateral spinal flexion reflex. The Journal of Physiology.

[bib4] Gerstmann K, Jurčić N, Blasco E, Kunz S, de Almeida Sassi F, Wanaverbecq N, Zampieri N (2022). The role of intraspinal sensory neurons in the control of quadrupedal locomotion. Current Biology.

[bib5] Hubbard JM, Böhm UL, Prendergast A, Tseng PEB, Newman M, Stokes C, Wyart C (2016). Intraspinal sensory neurons provide powerful inhibition to motor circuits ensuring postural control during locomotion. Current Biology.

[bib6] Jalalvand E, Robertson B, Wallén P, Grillner S (2016). Ciliated neurons lining the central canal sense both fluid movement and ph through ASIC3. Nature Communications.

[bib7] Nakamura Y, Kurabe M, Matsumoto M, Sato T, Miytashita S, Hoshina K, Kamiya Y, Tainaka K, Matsuzawa H, Ohno N, Ueno M (2023). Cerebrospinal fluid-contacting neuron tracing reveals structural and functional connectivity for locomotion in the mouse spinal cord. eLife.

[bib8] Orts-Del’Immagine A, Cantaut-Belarif Y, Thouvenin O, Roussel J, Baskaran A, Langui D, Koëth F, Bivas P, Lejeune F-X, Bardet P-L, Wyart C (2020). Sensory neurons contacting the cerebrospinal fluid require the reissner fiber to detect spinal curvature in vivo. Current Biology.

[bib9] Sternberg JR, Prendergast AE, Brosse L, Cantaut-Belarif Y, Thouvenin O, Orts-Del’Immagine A, Castillo L, Djenoune L, Kurisu S, McDearmid JR, Bardet PL, Boccara C, Okamoto H, Delmas P, Wyart C (2018). Pkd2l1 is required for mechanoception in cerebrospinal fluid-contacting neurons and maintenance of spine curvature. Nature Communications.

[bib10] Wu MY, Carbo-Tano M, Mirat O, Lejeune FX, Roussel J, Quan FB, Fidelin K, Wyart C (2021). Spinal sensory neurons project onto the hindbrain to stabilize posture and enhance locomotor speed. Current Biology.

[bib11] Wyart C, Del Bene F, Warp E, Scott EK, Trauner D, Baier H, Isacoff EY (2009). Optogenetic dissection of a behavioural module in the vertebrate spinal cord. Nature.

